# Perceived Produce Availability and Child Fruit and Vegetable Intake: The Healthy Communities Study

**DOI:** 10.3390/nu13113681

**Published:** 2021-10-20

**Authors:** Laurel F. Moffat, Lorrene D. Ritchie, Wendi Gosliner, Kaela R. Plank, Lauren E. Au

**Affiliations:** 1 Youth and Families Unit, Extension, Washington State University, Spokane, WA 99202, USA; laurel.moffat@wsu.edu; 2 Nutrition Policy Institute, Division of Agriculture and Natural Resources, University of California, Oakland, CA 94607, USA; lritchie@ucanr.edu (L.D.R.); wgosliner@ucanr.edu (W.G.); krplank@ucanr.edu (K.R.P.); 3 Department of Nutrition, University of California, Davis, CA 95616, USA

**Keywords:** food environment, diet, fruits and vegetables, children

## Abstract

Children eat more fruits and vegetables when more are available at home, but less is known about how the neighborhood food environment relates to children’s diet and weight outcomes. The goal of this study was to determine whether parental perception of the food environment (neighborhood and home) is associated with children’s fruit and vegetable (F&V) intake and weight outcomes, and to assess differences by household food security status and household income. Cross-sectional data from the 2013–2015 U.S. Healthy Communities Study included 5138 children, aged 4 to 15 years old, from 130 U.S. communities. Neighborhood and home food environments were assessed with parent-reported, perceived F&V availability scores. Associations were tested with multi-level linear regression models. Parents’ perception of produce availability was associated with household F&V availability ratings (β = 0.09 points, *p* < 0.001). Household F&V availability was associated with child F&V intake (β = 0.32 cups/day or 25.6 g/day, *p* < 0.001). A higher child F&V intake was associated with a lower child BMI z-score (β = −0.05, *p* = 0.002). Weaker relationships were seen for children living in food insecure or low-income households. Optimizing neighborhood and home access to F&V may help children improve diet quality, but may not be as effective for children living in food insecure or low-income households.

## 1. Introduction

Very few children in the United States (U.S.) meet the recommended daily intake for produce: less than 50% of children consume enough fruits, and less than 12% consume enough vegetables [1]. A low intake of fruits and vegetables has been linked to obesity, hypertension, coronary heart disease, stroke, and cancer [2]. Many factors contribute to low fruit and vegetable intake and associated chronic diseases, which is why the American Academy of Pediatrics (AAP) and the Centers for Disease Control and Prevention recommend a multi-level approach to establishing healthy dietary patterns and preventing obesity, addressing the neighborhood and home food environments in addition to individual-level dietary factors [3,4]. However, the nature of the relationships between food environments, children’s fruit and vegetable intake, and weight status requires further investigation to identify the most promising strategies to optimize child health.

Strong evidence suggests that food availability in the home is associated with children’s fruit and vegetable intake, which is supported by three systematic reviews that found positive associations between the availability of fruits and vegetables inside the home and child produce intake [5,6,7]. Evidence is weaker for the association between children’s fruit and vegetable intake and body mass index (BMI), but the AAP recommends including increased produce consumption in obesity prevention strategies for its low calorie, high nutrient density, and other health benefits [4]. The role of the neighborhood food environment in children’s fruit and vegetable intake is also unclear. In the one review that assessed neighborhood environment and diet, researchers found a moderately strong relationship, but called for additional studies and better validation of environmental measures [8]. Studies of food environment interventions, such as corner store conversions and the introduction of grocery stores, have found minor improvements in fruit and vegetable intake [9]. While previous studies of children’s diets and BMI have focused on either neighborhood or home food environments, few studies have examined the relationships between all four levels: the neighborhood food environment, home food environment, child eating behavior, and child body weight.

Many factors may influence the relationship between neighborhood and home food environments and the dietary intakes of children, but few studies have tried to quantify interactions with household income and food security, which is characterized by a lack of reliable access to adequate food [10]. Previous studies have shown an association between household food security and income with reduced in-home produce availability and fruit consumption, but studies have reported conflicting findings related to whether food security status moderates the relationship between food environments and diet [11,12,13]. A 2019 review of the associations between food environments and diet by household income found some evidence that a low household income is associated with stronger relationships between food environments and diet, when measuring neighborhood environments using food pricing; one study that examined these relationships using perceived food environments found that adolescents from lower-income families were more responsive to a negative food environment (greater access to fast food and convenience stores was associated with an unhealthier diet) [14].

The primary aim of this study was to determine whether there were associations between parents’ perceptions of produce availability in their neighborhood, household fruit and vegetable availability, child fruit and vegetable intake, and child BMI z-score. The hypothesis was that higher perceptions of neighborhood produce availability by parents would be associated with higher household produce availability, higher child consumption of fruits and vegetables, and lower child BMI. A secondary aim was to determine whether any significant relationships in these variables varied by household food security status or annual household income. The hypothesis was that children living in food insecure and low-income households would have lower household fruit and vegetable availability and consumption, and higher BMI z-scores than children living in food secure and higher income households, regardless of parent perceptions of neighborhood produce availability.

## 2. Materials and Methods

### 2.1. Study Design

The 2013–2015 Healthy Communities Study measured associations among community programs and policies and obesity-related outcomes in a sample of 5138 children aged 4–15 years old in the United States [15]. The study was observational and used clustered sampling by community and by school. Communities, defined as high school catchment areas, were selected either because they had known child obesity interventions (n = 28) or through national probability-based sampling (n = 102). For the latter, communities were stratified by region of the U.S., urbanicity, race/ethnicity, and income, then sampled with probabilities proportional to the number of children aged 4–15 years living in the community [16]. Up to two public elementary and two public middle schools were selected from each community, and children were recruited from each school [17].

Children and adult caregivers (hereafter referred to as parents) were interviewed in their homes by trained data collectors [17]. For questions asked of children, parents were the primary respondent if children were under 8 years old; otherwise, parents were present to assist if needed [18]. Parents were the primary respondents for questions about the household. All procedures involving research study participants were approved by the Battelle Memorial Institute IRB. Written informed consent was obtained from all parents, and children aged 8 years or older provided written assent.

### 2.2. Measures

For the perceived neighborhood availability of produce (i.e., fresh fruits and vegetables), parents were asked to rate how much they agreed with statements that there is a large selection of produce, it is high quality, and it is easy to purchase (1—disagree a lot, 2—disagree a little, 3—agree a little, or 4—agree a lot). Responses to these three questions were averaged and combined into one composite score for perceived neighborhood produce availability. For household fruit and vegetable availability, parents were asked how often fruits and dark green vegetables were typically available in their home (0—never, 1—rarely, 2—sometimes, 3—often, or 4—very often), and the responses to these two questions were averaged.

Child fruit and vegetable intake was measured with a Dietary Screener Questionnaire developed by the National Cancer Institute [19]. Daily fruit and vegetable intake in cups/day (1 cup = 80 g) was calculated based on fruit, vegetable, and legume consumption frequency over the past month, including 100% fruit juice and excluding fried potatoes [18]. Fried potatoes, but not baked, boiled, or other potatoes, were excluded from the fruit and vegetable calculation because they are considered energy-dense foods of minimal nutritional value [18]. Using a protocol developed by the National Cancer Institute, nationally representative sex- and age-specific mean daily intakes from 24-h recalls were used to convert frequencies into cups per day consumed. Child height and weight were measured using standard protocols, and age- and sex-adjusted BMI z-scores were calculated using the 2000 CDC Growth Charts [20]. Household food insecurity status was determined using a validated subset of two items from the U.S. Department of Agriculture’s Food Security Survey Modules [21]. Parents reported household food insecurity (often true, sometimes true, or never true) based on two questions: (1) “Within the past 12 months we worried whether our food would run out before we got money to buy more” and (2) “Within the past 12 months the food we bought just did not last and we did not have money to get more”. Households were classified as food insecure if responses were affirmative (often or sometimes) to one or both items. Annual household income was based on the self-reported income level. Other demographic factors were measured via parent survey questions, and community information was collected for census tracts from the 2009–2013 American Community Survey [16].

### 2.3. Data Analysis

Missing data were addressed using multiple imputation. Multilevel linear regression models adjusted for random effects (community and school) and covariates were used to test associations between variables of interest:

- Model 1: perceived neighborhood produce availability score (predictor) in relation to household fruit and vegetable availability score (outcome).
- Model 2: perceived neighborhood produce availability score (predictor) in relation to child fruit and vegetable intake (outcome).
- Model 3: household fruit and vegetable availability score (predictor) in relation to child fruit and vegetable intake (outcome).
- Model 4: perceived neighborhood produce availability score (predictor) in relation to child BMI z-score (outcome).
- Model 5: household fruit and vegetable availability score (predictor) in relation to child BMI z-score (outcome).
- Model 6: child fruit and vegetable intake (predictor) in relation to child BMI z-score (outcome).

Covariates for models with child fruit and vegetable intake and BMI z-score as outcomes were selected using the least absolute shrinkage and selection operator (LASSO) method [22]. Covariates for models with child fruit and vegetable intake as the outcome included the following: annual household income, maximum parent education, maximum parent employment, child race, child ethnicity, season of the year, region of the U.S., community percent minority, community urbanicity, community percent African American, community percent Hispanic, community percent in poverty, and community percent unemployed. The model with the household fruit and vegetable availability score as the outcome used the same covariates. Covariates for models with child BMI z-score as the outcome included the following: annual household income, child height, paternal education, maternal employment, child ethnicity, region of the U.S., and community percent minority. Significant associations were then tested for interaction by household food insecurity status and by annual household income. Analyses were performed using SAS 9.4 (SAS Institute, Cary, NC, USA) with significance determined by *p* < 0.05.

## 3. Results

The average age of children in this sample was 9 years old (Table 1). Approximately half of the children were female, and just under half (45%) were Hispanic. Forty-one percent of children were classified as overweight or obese based on having a BMI above the age- and sex-specific 85th percentile. Forty-three percent of parents had a maximum education level of high school or less, and in most households (73%) at least one parent was working full time. Forty-five percent of the households surveyed had experienced food insecurity in the past year, and half of the households had total annual incomes of $35,000 or less. The children lived in communities that varied in terms of location, but were most often located in the South (42%) and were classified as suburban (40%) or urban (38%). Communities on average were one-third Hispanic or Latino and one-fifth African American, and had a poverty rate of 21%. On average (mean ± SD), parents rated the produce availability in their communities as 3.4 (±0.8) out of 4 points (with a score of 4 signifying high agreement with large selection, high quality, and ease of purchasing statements). The mean household fruit and vegetable availability score was 3.3 ± 0.7 out of 4 points (with a score of 4 signifying frequent availability of both fruits and dark green vegetables). On average, children consumed 2.5 ± 0.9 cups of fruits and vegetables per day and had a BMI z-score of 0.7 ± 1.2. Perceived neighborhood produce availability scores varied by region of the U.S., community urbanicity, household annual income, and household food insecurity status, with the highest average scores in the Western region (3.5 ± 0.7 points, *p* < 0.001, data not shown), suburban communities (3.5 ± 0.7, *p* < 0.001, data not shown), households earning more than $35,000 (3.5 ± 0.7, *p* < 0.001, data not shown), and food secure households (3.5 ± 0.7, *p* < 0.001, data not shown).

Parents’ perception of produce availability in their neighborhood was positively associated with household fruit and vegetable availability ratings (Table 2). Each one-point increase in perceived neighborhood produce availability score was associated with a 0.09-point higher average household fruit and vegetable availability score (95% CI: 0.06, 0.11). The perceived neighborhood produce availability score was not associated with the children’s average fruit and vegetable intake or children’s average BMI z-score. Parents’ rating of household fruit and vegetable availability was positively associated with children’s fruit and vegetable intake. Each one-point increase in household fruit and vegetable availability score was associated with a 0.32 cup increase children’s average daily fruit and vegetable consumption (95% CI: 0.28, 0.35). The household-level availability score was not associated with the children’s average BMI z-score. Fruit and vegetable intake by children was negatively associated with the child BMI z-score. Each additional cup of fruits and vegetables consumed by children was associated with a 0.05 lower child BMI z-score on average (95% CI: −0.09, −0.02).

The association between the perceived neighborhood produce availability score and household fruit and vegetable availability did not vary by household food security status (p-interaction = 0.05) (Table 3). The association between household fruit and vegetable availability score and child fruit and vegetable intake varied by food insecurity status (p-interaction = 0.03). Among food insecure households, a one-point increase in household fruit and vegetable availability score was associated with a smaller increase in average child fruit and vegetable intake (0.28 cups/day, 95% CI: 0.23, 0.33) compared with food secure households (0.36 cups/day; 95% CI: 0.30, 0.42). The association between child fruit and vegetable intake and child BMI z-score also varied by food security status (p-interaction = 0.01). Among children in food insecure households, increased fruit and vegetable intake was not associated with a change in the average BMI z-score (95% CI: −0.05, 0.05). Among children in food secure households, each additional cup of fruits and vegetables consumed per day was associated with a 0.10 lower average BMI z-score (95% CI: −0.14, −0.05).

The association between perceived neighborhood produce availability and household fruit and vegetable availability scores did not vary by household income (p-interaction = 0.80) (Table 4). Low-income households compared to high-income households had a smaller average increase in child fruit and vegetable intake for every one-point increase in household fruit and vegetable availability (0.28 cups/day vs. 0.37 cups/day, respectively; p-interaction = 0.02). The association between child fruit and vegetable intake and average child BMI z-score did not vary by household income (p-interaction = 0.33).

## 4. Discussion

Improved perception of produce availability at the neighborhood-level was associated with a higher produce availability at the household-level, which in turn was related to improved fruit and vegetable intake at the individual-level. Higher fruit and vegetable intake among children was associated with a lower BMI, which has been found in previous studies with adults [23,24,25,26], but has been shown with mixed results in children [27]. These results are consistent with reviews that found positive associations between home food availability and child produce intake [5,6,7], and further support the connection between the perceived neighborhood food environment and the home food environment. The associations between produce availability, intake, and BMI suggest a pathway between neighborhood-level, household-level, and individual-level factors that is consistent with the study hypothesis that the four factors are related. These connections between community, household, and individual factors are also consistent with the IOM’s multilevel model of eating behavior and body weight determinants [28].

The findings from this study align with those observed in the 2008–2010 INPACT study of 1501 Dutch families, which found that parents’ perceptions of the neighborhood food environment were associated with produce availability at home, but neighborhood measures were not associated with children’s fruit and vegetable intake [29]. The results are also consistent with the M-TEENs study of 903 U.S. adolescents, which found associations between home food availability and children’s diet, but no connections between the neighborhood food environment and children’s diet and BMI [30]. It is likely that associations across multiple levels (neighborhood availability relating to children’s intake or household availability relating to children’s weight status) were not seen because additional factors at each level combine with the effects of food environments to predict eating behaviors and weight status. For example, parents’ eating preferences, behaviors, and encouragement could be just as predictive of child intake as home produce availability [5,6,7,30,31,32,33,34]. Therefore, this study’s results confirm that food environments play an important but not exclusive role in children’s fruit and vegetable consumption.

Household food security status and annual income modified some associations. The associations between household produce availability and child fruit and vegetable intake and between child fruit and vegetable intake and child BMI z-score had smaller effect sizes among children living in food insecure households. These results are consistent with a 2016 study of barriers to fruit and vegetable consumption among 531 adults from Oakland, CA, in which food insecurity was associated with weaker or nonsignificant associations between barriers and consumption, suggesting that food insecurity may attenuate the effects of other factors on consumption [13]. Annual household income had a similar interaction on the association between household produce availability and child fruit and vegetable intake. Among children living in low-income households, greater availability was associated with smaller improvements in intake, which is in contrast to a previous review that showed stronger associations between food environment and diet among low-income participants when the food environment was measured using food prices [14].

The strengths of this study include that this was one of the first national studies to include both the neighborhood and home food environments in an analysis of children’s fruit and vegetable intake, and it examined how food security and income modified these relationships. Furthermore, the Healthy Communities Study had a large sample size and high proportion of participants who reported household food insecurity or low annual income relative to similar studies, improving the power in these subgroups.

Limitations of the study include that availability and diet measures were self-reported, which increased the risk of reporting and recall bias by sociodemographic factors [8,35]. Neighborhood produce availability was measured based on adults’ perceptions instead of objective assessment, although there is some evidence that the perceived availability better captures the true experience of food accessibility because respondents will consider safety, transportation access, and other experiential factors in their assessment of food access [8]. Household food availability was also assessed using parent self-report, and measures of vegetable availability were limited to only dark green vegetables. The neighborhood availability score included “ease of purchasing” instead of perceptions of food prices or “affordability”, both of which are known to affect perceived food availability and purchasing behavior [36].

The findings from this study suggest that interventions may not find direct relationships between changes in local food environments and children’s fruit and vegetable intake or BMI, and that the household-level food environment and household food insecurity and income may play important intermediary roles in the relationship between these factors. Further analyses should explore the role of household fruit and vegetable availability as a mediator between perceived neighborhood produce availability and child fruit and vegetable intake. Food environment interventions to increase the access, variety, and quality of fruits and vegetables in a neighborhood should consider the household-level food environment, household food security status, and household income when assessing impacts on children’s fruit and vegetable intake.

## Figures and Tables

**Table 1:** Characteristics of children, households, and communities in the Healthy Communities Study (N = 5138 children, 130 communities).

Child and Household Characteristics	*n*	%
Female child	2614	50.9
Child race/ethnicity		
Hispanic or Latino	2295	44.7
Non-Hispanic White	1520	29.6
Non-Hispanic African American	927	18.0
Non-Hispanic Other	396	7.7
Child overweight or obese	2081	40.5
Household food insecure	2293	44.6
Household annual income		
Up to $35,000	2640	51.4
Greater than $35,000	2498	48.6
Maximum parent education level for biological parents		
Less than high school	1166	22.7
High school diploma, GED or equivalent	1038	20.2
Some college or Associate Degree	1284	25.0
Bachelor’s Degree	786	15.3
Graduate Degree	868	16.9
Maximum employment status of biological parents		
Working full-time for pay	3747	72.9
Working part-time for pay	518	10.1
Unemployed	313	6.1
Other	539	10.5
**Community Characteristics**		
U.S. region		
Midwest	991	19.3
Northeast	791	15.4
South	2135	41.6
West	1221	23.8
Urbanicity		
Rural	1162	22.6
Suburban	2034	39.6
Urban	1942	37.8
**Child and Household Characteristics**	**Mean**	**SD**
Child age (years)	9.3	2.7
**Community Characteristics**		
Community race/ethnicity		
Percent of population aged 5 to 14 years that are African American	19.7	23.4
Percent of population aged 5 to 14 years that are Hispanic	34.7	29.6
Community socioeconomic status		
Percent of population below the federal poverty level	20.6	10.6
Percent of population in labor force 16 years and over who are unemployed	8.8	3.4
**Outcomes**		
Perceived neighborhood produce availability score ^1^	3.4	0.8
Ease of access ^1^	3.5	0.8
Variety ^1^	3.4	0.9
Quality ^1^	3.2	0.9
Household fruit and vegetable availability score ^2^	3.3	0.7
Fruit availability ^2^	3.1	0.9
Dark green vegetable availability ^2^	3.5	0.7
Child fruit and vegetable intake (cups/day) ^3^	2.5	0.9
Body-mass-index-for-age-z-score (BMI z-score)	0.7	1.2

^1^ Questions on neighborhood produce (ease of purchasing, large selection, and high quality) were scored as follows: 1—disagree a lot, 2—disagree a little, 3—agree a little, or 4—agree a lot. ^2^ Questions on the frequency of the availability of fruits and dark green vegetables at home were scored as follows: 0—never, 1—rarely, 2—sometimes, 3—often, or 4—very often. ^3^ One cup is approximately equivalent to 80 g of fruits and vegetables.

**Table 2:** Associations between perceived neighborhood produce availability and household fruit and vegetable availability, child fruit and vegetable intake, and child body mass index z-score (BMI z-score) in the Healthy Communities Study (N = 5138).

Model	Predictor	Outcome	β	95% CI	*p*
1	Perceived neighborhood produce availability score ^1^	Household fruit and vegetable availability score ^2,3^	0.09	0.06, 0.11	<0.001
2	Perceived neighborhood produce availability score ^1^	Child fruit and vegetable intake (cups/day) ^3^	0.03	0.00, 0.07	0.08
3	Household fruit and vegetable availability score ^2^	Child fruit and vegetable intake (cups/day) ^3^	0.32	0.28, 0.35	<0.001
4	Perceived neighborhood produce availability score ^1^	Child BMI z-score ^4^	−0.03	−0.07, 0.02	0.25
5	Household fruit and vegetable availability score ^2^	Child BMI z-score ^4^	0.00	−0.05, 0.04	0.83
6	Child Fruit and vegetable intake (cups/day)	Child BMI z-score ^4^	−0.05	−0.09, −0.02	0.002

^1^ Questions on neighborhood produce (ease of purchasing, large selection, and high quality) were scored as follows: 1—disagree a lot, 2—disagree a little, 3—agree a little, or 4—agree a lot. ^2^ Questions on frequency of availability of fruits and dark green vegetables at home were scored as follows: 0—never, 1—rarely, 2—sometimes, 3—often, or 4—very often. ^3^ Models 1–3 are adjusted for the following covariates: annual household income, maximum parent education, maximum parent employment, child race, child ethnicity, season of the year, region of the U.S., percent minority in census tract, community urbanicity, community percent African American, community percent Hispanic, community percent in poverty, and community percent unemployed. ^4^ Models 4–6 are adjusted for the following covariates: annual household income, child height, paternal education, maternal employment, child ethnicity, region of the U.S., and percent minority in census tract.

**Table 3:** Select relationships between perceived neighborhood produce availability, household fruit and vegetable availability, child fruit and vegetable intake, and child body mass index z-score (BMI z-score) in the Healthy Communities Study, by household food security status (N = 5138).

		Food Insecure Households ^1^ (n = 2293)		Food Secure Households ^1^ (n = 2845)		
Predictor	Outcome	β	95% CI	β	95% CI	*p* Interaction
Perceived neighborhood produce availability score ^2^	Household fruit and vegetable availability score ^3,4^	0.10	0.07, 0.14	0.06	0.02, 0.09	0.05
Household fruit and vegetable availability score ^3^	Child fruit and vegetable intake (cups/day) ^4^	0.28	0.23, 0.33	0.36	0.30, 0.42	0.03
Child Fruit and vegetable intake (cups/day)	Child BMI z-score ^5^	0.00	−0.05, 0.05	−0.10	−0.14, −0.05	0.01

^1^ Food insecurity status was determined using a validated two-item screener. ^2^ Questions on neighborhood produce (ease of purchasing, large selection, and high quality) were scored as follows: 1—disagree a lot, 2—disagree a little, 3—agree a little, or 4—agree a lot. ^3^ Questions on frequency of the availability of fruits and dark green vegetables at home were scored as follows: 0—never, 1—rarely, 2—sometimes, 3—often, or 4—very often. ^4^ Models are adjusted for the following covariates: household food security status, perceived neighborhood produce availability score, annual household income, maximum parent education, maximum parent employment, child race, child ethnicity, season of the year, region of the U.S., community urbanicity, percent minority in census tract, community percent African American, community percent Hispanic, community percent in poverty, and community percent unemployed. ^5^ Model is adjusted for the following covariates: household food security status, child fruit and vegetable intake, annual household income, child height, paternal education, maternal employment, child ethnicity, region of the U.S., and percent minority in census tract.

**Table 4:** Select relationships between perceived neighborhood produce availability, household fruit and vegetable availability, child fruit and vegetable intake, and child body mass index z-score (BMI z-score) in the Healthy Communities Study, by annual household income (N = 5138).

		Low-Income Households ^1^ (n = 2640)		High-Income Households ^1^ (n = 2498)		
Predictor	Outcome	β	95% CI	β	95% CI	*p* Interaction
Perceived neighborhood produce availability score ^2^	Household fruit and vegetable availability score ^3,4^	0.09	0.06, 0.12	0.09	0.05, 0.12	0.80
Household fruit and vegetable availability score ^3^	Child fruit and vegetable intake (cups/day) ^4^	0.28	0.23, 0.33	0.37	0.31, 0.43	0.02
Child fruit and vegetable intake (cups/day)	Child BMI z-score ^5^	−0.04	−0.09, 0.01	−0.07	−0.13, −0.02	0.33

^1^ Households were considered low-income if parents reported annual household earnings less than $35,000. ^2^ Questions on neighborhood produce (ease of purchasing, large selection, and high quality) were scored as follows: 1—disagree a lot, 2—disagree a little, 3—agree a little, or 4—agree a lot. ^3^ Questions on the frequency of the availability of fruits and dark green vegetables at home were scored as follows: 0—never, 1—rarely, 2—sometimes, 3—often, or 4—very often. ^4^ Models are adjusted for the following covariates: perceived neighborhood produce availability score, maximum parent education, maximum parent employment, child race, child ethnicity, season of the year, region of the U.S., community urbanicity, percent minority in census tract, community percent African American, community percent Hispanic, community percent in poverty, and community percent unemployed. ^5^ Model is adjusted for the following covariates: child fruit and vegetable intake, child height, paternal education, maternal employment, child ethnicity, region of the U.S., and percent minority in census tract.

## Data Availability

Data are available in a publicly accessible repository that does not issue DOIs. Publicly available datasets were analyzed in this study. This data can be found here: https://biolincc.nhlbi.nih.gov/studies/hcs/ (accessed on 18 October 2021).
